# Modulation of the extracellular matrix patterning of thrombospondins by actin dynamics and thrombospondin oligomer state

**DOI:** 10.1042/BSR20140168

**Published:** 2015-06-22

**Authors:** Andrew L. Hellewell, Xianyun Gong, Karsten Schärich, Elena D. Christofidou, Josephine C. Adams

**Affiliations:** * School of Biochemistry, University of Bristol, Bristol BS8 1TD, U.K.

**Keywords:** actin, coiled-coil, cytochalasin D, extracellular matrix, jasplakinolide, thrombospondins

## Abstract

Thrombospondins (TSPs) are evolutionarily-conserved, secreted glycoproteins that interact with cell surfaces and extracellular matrix (ECM) and have complex roles in cell interactions. Unlike the structural components of the ECM that form networks or fibrils, TSPs are deposited into ECM as arrays of nanoscale puncta. The cellular and molecular mechanisms for the patterning of TSPs in ECM are poorly understood. In the present study, we investigated whether the mechanisms of TSP patterning in cell-derived ECM involves actin cytoskeletal pathways or TSP oligomer state. From tests of a suite of pharmacological inhibitors of small GTPases, actomyosin-based contractility, or actin microfilament integrity and dynamics, cytochalasin D and jasplakinolide treatment of cells were identified to result in altered ECM patterning of a model TSP1 trimer. The strong effect of cytochalasin D indicated that mechanisms controlling puncta patterning depend on global F-actin dynamics. Similar spatial changes were obtained with endogenous TSPs after cytochalasin D treatment, implicating physiological relevance. Under matched experimental conditions with ectopically-expressed TSPs, the magnitude of the effect was markedly lower for pentameric TSP5 and *Drosophila* TSP, than for trimeric TSP1 or dimeric *Ciona* TSPA. To distinguish between the variables of protein sequence or oligomer state, we generated novel, chimeric pentamers of TSP1. These proteins accumulated within ECM at higher levels than TSP1 trimers, yet the effect of cytochalasin D on the spatial distribution of puncta was reduced. These findings introduce a novel concept that F-actin dynamics modulate the patterning of TSPs in ECM and that TSP oligomer state is a key determinant of this process.

## Abstract (precis)

We report that the patterning of thrombospondin (TSP) deposition into the extracellular matrix (ECM) is modulated by actin filament dynamics. Whereas actin-dependence of patterning is evolutionarily conserved, the extent of modulation correlates inversely with the oligomer state of the TSP.

## INTRODUCTION

Thrombospondins (TSPs) are an evolutionarily conserved family of large, calcium-binding glycoproteins. Designated as matricellular proteins, they are secreted and perform a diverse range of functions through interactions with the extracellular matrix (ECM), the cell surface and, indirectly, with the actin cytoskeleton [1–6]. TSPs have a substantial clinical significance with associations with both genetic and acquired human diseases. For example, mutations in TSP5 are causal in pseudoachrondroplasia (PSACH) [7–9] and single nucleotide polymorphisms (SNPs) identified in TSP1 and 4 have been linked to familial cardiovascular diseases [10–12]. In addition to familial diseases, the up-regulation of TSP1 or TSP2 has been shown to inhibit tumour growth through suppression of angiogenesis [13,14]. TSPs perform many of their roles through interaction with, and deposition into, the ECM and the presence of TSPs in ECM *in vivo* is well documented. For example TSP1 or TSP2 are up-regulated in ECM during wound repair, fibrotic responses and tumorigenesis and have roles in angiogenesis, platelet aggregation and the inflammatory response [1,13]. TSP5 is largely expressed in connective tissue and functions in cartilage ECM assembly [1,15].

Mammalian TSPs consist of five family members (TSP1–TSP5) of two sub-types, either homo-trimers (TSP1, TSP2) or homo-pentamers (TSP3, TSP4, TSP5) [1,16]. Although containing variable N-terminal regions, mammalian TSPs share common C-terminal regions that consist of three or four EGF-like domains, a series of calcium-binding type 3 repeats and a globular L-lectin-like domain [16–19]. The deposition of TSP1 into ECM is mediated by its C-terminal region in trimeric form [20]. ECM retention is a conserved property of TSPs and, for the TSPs analysed to date, involves the deposition of characteristic insoluble puncta into cell-derived ECM. These punctate structures are relevant to the *in vivo* ECM patterning of TSPs [20].

Extracellular TSP1 is known to regulate actin microfilament (F-actin) organization in multiple ways that promote cell motility [21]. TSP1 regulates the adherence of fibroblasts and endothelial cells to a fibronectin ECM through the disassembly of focal adhesions and F-actin bundles [22]. Soluble TSP1 regulates the phosphoinositol 3-kinase and cyclic-GMP-dependent kinase-dependent release of α-actinin and vinculin from adhesion plaques, a property that weakens cell–ECM attachment [23,24]. Cell spreading and migration on TSP1 substrata is associated with the formation of extensive lamellipodia [25]. TSP1 modulates F-actin organization through the actin-bundling protein fascin-1, leading to the formation of large lamellipodia with ribs, microspikes and filopodia that contain fascin-1 [26,27].

In contrast, very little is known about the mechanism for deposition of TSP puncta into ECM or if the actin cytoskeleton participates in these mechanisms. Nevertheless, a role for the actin cytoskeleton appears possible. For other ECM components such as fibronectin, extracellular fibril formation depends on actomyosin-based contractility [28–32]. Elevated ECM deposition of TSP1 or TSP2 is associated with situations of increased tissue stiffness, such as in fibrosis or atherosclerotic plaques; in these contexts, intracellular tensile forces are also increased [33]. The β1 integrin ECM adhesion receptors, which are linked indirectly to F-actin, are not essential for ECM deposition of TSP1, but increase the efficiency of its accumulation in ECM [20].

Our goal in the present study was to establish whether the actin cytoskeleton participates in the mechanism of TSP deposition into ECM and to analyse the contributions of various actin cytoskeletal pathways. We establish for the first time that F-actin integrity is important for the patterning of TSP puncta and demonstrate the relative importance of TSP oligomer state compared with protein sequence in this activity. These studies advance knowledge of the molecular and cell-dependent processes that modulate ECM accumulation of TSPs.

## EXPERIMENTAL

### Materials and cell culture

Membrane-permeable C3 transferase was from Cytoskeleton, Inc. Cytochalasin D, nocodazole, lysophosphatidic acid and FITC-phalloidin were from Sigma. All other pharmacological reagents were from Calbiochem. The following mouse monoclonal antibodies were used: to V5 epitope (GKPIPNPLLGLDST; [34]; Invitrogen); to FLAG epitope (M2 clone; Sigma); to DsRed (Clontech or Abcam); to β-tubulin (Sigma); to TSP1 (A6.1; Neomarkers). Rabbit monoclonal to COMP (EPR6289(2)) was from Abcam, FITC-conjugated goat polyclonal IgG to V5 tag was from Abcam. Alkaline phosphatase-conjugated secondary antibodies were from Applied Biosystems and FITC-conjugated secondary antibodies were from Sigma. COS7, SW480 human colon carcinoma and rat chondrosarcoma (RCS) cells (A.T.C.C.) were all cultured in Dulbecco's modified Eagle's medium (DMEM) containing 10% fetal calf serum (FCS) in a humidified, 5% CO_2_ incubator at 37°C.

### Thrombospondin expression plasmids

pCEP-pu plasmid was as described [17]. mRFPovTSP1C (monomeric RFP), TSP1.V5, TSP5.V5, D-TSP.V5 and Ci-TSPA.V5 were as described [20]. p3XFLAG-*Myc*-CMV-23 plasmid containing a Met-preprotrypsin leader sequence was obtained from Sigma–Aldrich. Oligonucleotide primers used to generate additional constructs within this plasmid and pCEP-pu are listed in Table 1 and were synthesized by Sigma–Aldrich.

**Table 1:** DNA oligonucleotide primers used in the present study

Oligonucleotides	DNA Sequence 5′–3′
TSP5 coiled coil	
690F	GATCAAGCTTGGACAGGGCCAGAGC
644R	GTACGTTAACTAGGTTACCCAGGGGCCGCACGCTGGGTAGGCC
TSP1C	
445F	GTACGTTAACATTGATGGATGCCTGTCCAAT
446R	GTACCTCGAGTTAGGGATCTCTACATTCGTA
TSP-5-1C	
690F	GATCAAGCTTGGACAGGGCCAGAGC
446R	GTACCTCGAGTTAGGGATCTCTACATTCGTA
FLAG–mRFP–TSP-5-1C	
787F	GATCGAATTCAATGGCCTCCTCC
779R	GTACTCTAGATTAGGGATCTCTACATTCGTATTTCAGG
FLAG–TSP1C	
688F	GATCAAGCTTATTGGCCACAAGACA
779R	GTACTCTAGATTAGGGATCTCTACATTCGTATTTCAGG
FLAG–TSP-5-1C	
690F	GATCAAGCTTGGACAGGGCCAGAGC
779R	GTACTCTAGATTAGGGATCTCTACATTCGTATTTCAGG

### Transfection and analysis of ECM accumulation

COS-7 cells (2 × 10^5^) were transiently transfected with 2.5 μg of the indicated plasmids using Polyfect transfection reagent (Qiagen) according to manufacturer's instructions. After overnight incubation, the cells from each transfection were replated on to glass coverslips in two replicate dishes or wells and incubated for 3 days to allow for secretion and deposition of ECM. One dish was then rinsed twice in 3× PBS and fixed in 2% paraformaldehyde (Electron Microscopy Sciences) for 10 min. These cells were used to quantify the percentage of cells expressing the protein. The parallel dish was treated with 20 mM ammonium hydroxide for 5 min to remove all cellular material except the insoluble ECM [35], rinsed 5× in double distilled H_2_O and fixed in 2% paraformaldehyde for 10 min. In experiments with inhibitors, 18–20 h after transfection, COS7 cells were replated on glass coverslips for 2 h and then treated with either 0.5 μM jasplakinolide, 5 μM cytochalasin D, 0.5 μg/ml paclitaxol or nocodazole, 230 ng/ml membrane permeable C3, 10 μM Y27362, 100 μM NSC23766, 10 ng/ml toxin B or 1 μg/ml LPA (lysophosphatidic acid) for 18 h before processing. SW480 and RCS cells were treated with 2.5 μM (SW480) or 1 μM (RCS) cytochalasin D, 2 and 24 h post-plating respectively. The concentrations of these agents were established in pilot dose-response experiments to perturb the relevant cytoskeletal system without preventing cell attachment or reducing cell viability over an 18 h period.

The coverslips from cultures expressing mRFP-tagged proteins were mounted directly on slides with Vectashield mounting medium containing DAPI (Vector Laboratories). Other samples were processed for indirect immunofluorescence before mounting. To verify the absence of cellular material after ECM isolation, samples were stained with DAPI as a nuclear stain. A minimum of two coverslips were scored for each sample in each experiment and a minimum of three independent experiments were carried out for each experimental condition.

### Analysis of TSP proteins by SDS/PAGE and immunoblot

TSP proteins were collected from conditioned media 2 days after transfection, using either RFP-Trap_A beads for RFP-tagged proteins (Chromotek) or immunoprecipitation with anti-FLAG monoclonal antibody M2 (Sigma) bound to rProtein G Agarose beads (Invitrogen) for the FLAG-tagged proteins. Thirty microlitre of packed bead slurry bound to 2 μg of antibody was incubated with 3 ml of conditioned media containing complete protease inhibitor cocktail (Roche) for 3 h at 4°C before centrifugation and washing three times in Tris-buffered saline containing 2 mM CaCl_2_. Proteins were eluted from the beads by boiling in SDS sample buffer with or without 100 mM DTT. SDS/PAGE under reducing or non-reducing conditions followed by immunoblotting was carried out as described [36].

### Fluorescence microscopy, scoring and image analysis

ECM samples for widefield fluorescence microscopy were examined under a Leica DMIRE2 inverted microscope (Heidelberg), equipped with electronically controlled shutters and filter wheel. Images were captured under a Leica HCX Pl Apo 63×/N. A. 1.32 Oil Ph 3 Cs objective lens using a Hamamatsu camera controller C4742_95 run by Leica Applications Systems software (version 3.1.5). Cellular samples were examined under a Leica SP5-AOBS confocal laser scanning microscope with HCX Plan Apo 100× N.A.1.4 oil immersion objective lens, using Leica confocal software version 2.5. Confocal images were acquired as XY sections at zoom 1, at room temperature. The percentage of transfected cells expressing the protein of interest was calculated in each experiment by direct scoring of cells expressing mRFP-tagged proteins as a fraction of DAPI-stained nuclei or by permeabilization in 0.5% Triton X-100 and indirect immunofluorescent staining with appropriate antibodies for the non-RFP-tagged proteins. Ten to twenty 63× fields were scored per coverslip. To quantify ECM retention of the various proteins, the total number of arrays of puncta/63× field and the number of arrays with an edge concentrated pattern of puncta, were scored from 10–30 fields per coverslip. For SW480 cells, individual arrays were scored from four separate experiments and four coverslips per experiment. In all experiments, the percentage of arrays with edge concentrated puncta was quantified. To quantify the pixel intensity across an array of puncta, a line was drawn across the centre of an array from a representative fluorescence image in ImageJ 1.46v (Figure 1B). Using the Plot Profile function, the pixel intensity along the line was measured and plotted against distance along the line. To compare between lines of different total lengths, the total length of the line for each array was normalized to 1.

**Figure 1:**
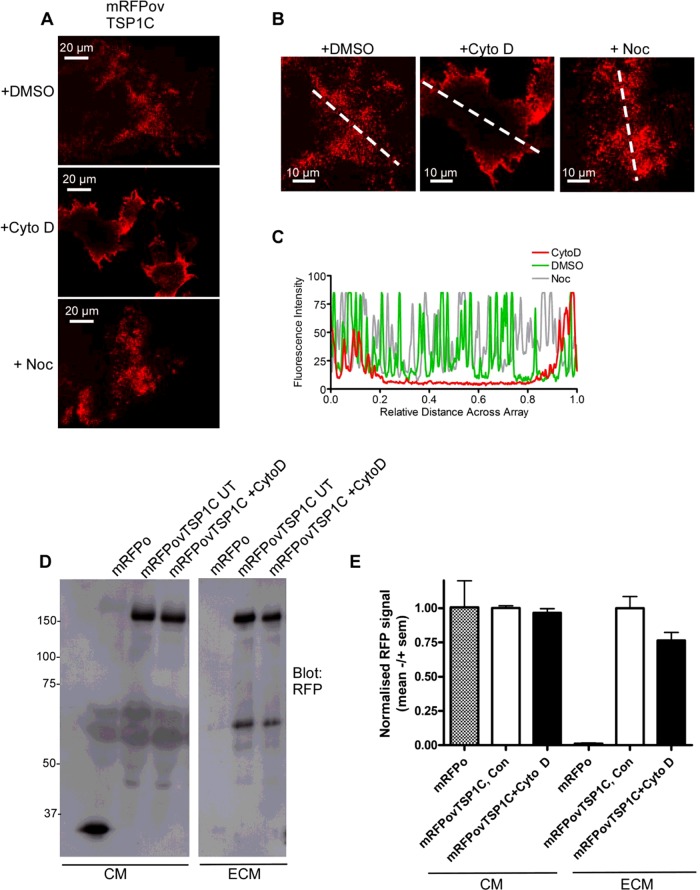
Effect of cytochalasin D and nocodazole on ECM patterning of mRFPovTSP1C (A) Representative fluorescence images of ECM deposition patterns of mRFPovTSP1C after treatment of cells with vehicle (DMSO), 0.5 μM cytochalasin D or 0.5 μg/ml nocodazole for 18 h. (**B**) Representative fluorescence images from (**A**) used to determine fluorescence intensity across array. Line used to measure relative distance across each array is shown. (**C**) Line scan of fluorescence intensity across fluorescence images shown in (**B**) using the plot profile function on ImageJ. (version 1.46r). To compare between images, the total line distance for each image was normalized to 1. (**D**) Immunoblots of TALON-pulldowns from conditioned media (CM) and ammonium hydroxide-extracted ECM, from an equal number of COS7 cells transfected with the indicated constructs, treated with either cytochalasin D or DMSO (UT). (**E**) Quantification of RFP signal from CM and ECM of COS7 cells transfected with the indicated constructs, from two immunoblots. Proteins were resolved on 10% SDS/PAGE gels under reducing conditions. Molecular mass markers are in kDa. Columns represent the mean and bars indicate S.E.M. from two independent experiments.

## RESULTS

### Identification of a role for F-actin in the patterning of TSP1 within ECM

TSPs are deposited into cell-derived ECM as arrays of fine puncta [20]. How this patterning is regulated by cells is not understood. To establish whether cytoskeletal organization contributes to the patterning of TSP1 within ECM, we tested the effects of pharmacological perturbations of the F-actin or microtubule cytoskeletons on the ECM retention of a model, fluorescently-tagged mini-trimer, mRFPovTSP1C. This engineered, trimeric protein contains the domains of TSP1 that are necessary and sufficient for ECM accumulation [20]. Because the goal of the experiments was to investigate effects on deposition into the insoluble ECM, pilot dose-response experiments established the minimum concentrations of the agents that were effective for perturbation of the relevant cytoskeletal system without preventing cell attachment or reducing cell viability over the time period needed for ECM accumulation of TSP1 to a sufficient level for quantified scoring, typically 18–24 h. In the final protocol to examine effects on patterning of newly deposited mRFPovTSP1C, cells were transfected, incubated for 18 h for protein expression, re-plated on to glass coverslips for 2 h for cell attachment and then incubated in the presence of the pharmacological agents for 18 h. Parallel samples were then processed either by fixation for scoring of cell numbers and cell morphology or by ammonium hydroxide extraction to isolate the insoluble cell-derived ECM [20].

Under control conditions, mRFPovTSP1C accumulates in ECM as arrays of relatively uniformly spaced small puncta (Figure 1A, +DMSO panel). Cytochalasin D that inhibits actin polymerization by binding to F-actin barbed ends [37], had a striking effect on the ECM patterning of mRFPovTSP1C puncta, such that puncta were deposited non-uniformly and concentrated around the edges of the puncta arrays (Figure 1A, +CytoD panel). In contrast, nocodazole, which depolymerizes microtubules, did not alter the patterning of puncta as compared with control conditions (Figure 1A, +Noc panel). The quantitative change in patterning after cytochalasin D treatment of cells was demonstrated by linescan plots of fluorescence intensity across the arrays, (examples shown in Figures 1B and 1C). Both inhibitors resulted in reduced cell spreading and each inhibitor had its expected effect on F-actin or microtubule integrity respectively (Supplementary Figure S1). Because nocodazole treatment also resulted in cell rounding but did not alter the pattern of deposition of mRFPovTSP1C, we infer that the effect of cytochalasin D was not a consequence of cell rounding *per se*.

Cytochalasin D did not reduce the amount of mRFPovTSP1C in the conditioned media of transfected cells (Figures 1D and 1E) nor did it alter the accumulation of mRFPovTSP1C in the ECM compared with the control condition (Figures 1D and 1E). A trimeric mRFPo control protein was secreted into the media but, as expected, did not accumulate in the ECM (Figure 1D and 1E). Thus, the altered patterning of mRFPovTSP1C within ECM appeared to result from a functional effect of altered actin cytoskeletal organization on post-secretion processes.

### Alterations in ECM patterning of mRFPovTSP1C are linked specifically to F-actin dynamics

To investigate the molecular basis for this action of cytochalasin D, additional pharmacological agents that perturb F-actin stress fibres or actin-associated proteins, including inhibitors or activators of members of the Rho family of small GTPases, the Rho/Rho kinases pathway and actomyosin contractility, were tested in comparison with cytochalasin D. Effects on puncta patterning in ECM were quantified from multiple experiments. None of the pharmacological agents markedly affected the expression of mRFPovTSP1C by the transfected cells (Figure 2A). Blebbistatin, an inhibitor of myosin ATPase or ML-7, an inhibitor of myosin light chain kinase, did not alter the patterning of mRFPovTSP1C within ECM (Figure 2B). Hyper-activation of Rho GTPase by treatment of cells with LPA, inhibition of Rho with membrane-permeable C3-transferase or inhibition of Rho kinases with Y27632, similarly did not alter the patterning of mRFPovTSP1C puncta (Figure 2B). *Clostridium difficile* Toxin B that inhibits multiple Rho family small GTPases [38] and NSC23766 that specifically inhibits Rac [39] did not alter the patterning of puncta (Figure 2B). It should be noted that some of these treatments reduced the level of mRFPovTSP1C present in the conditioned media (Figures 2C and 2D); however, in all cases, sufficient mRFPovTSP1C accumulated in ECM for effects on ECM patterning to be detected. Overall, these findings led us to focus specifically on mechanisms of global F-actin assembly and dynamics.

**Figure 2:**
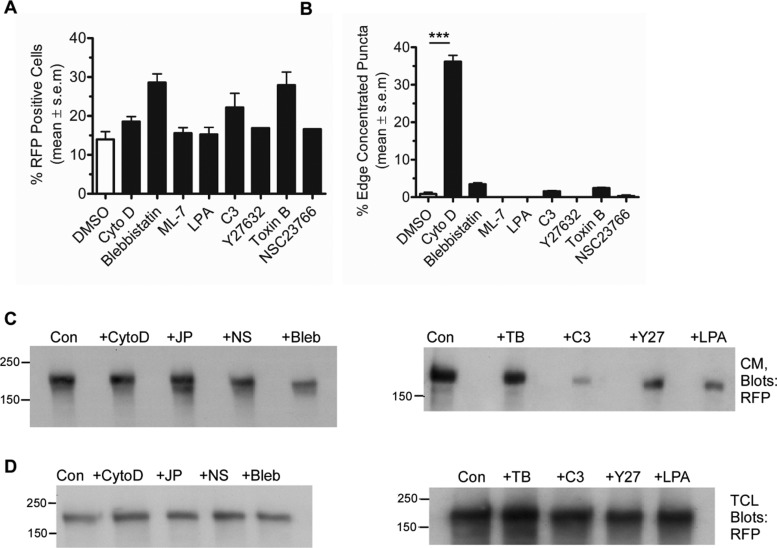
Effects of inhibitors of actin or actin-related pathways on ECM patterning of mRFPovTSP1C (**A**) Quantification of mRFPovTSP1C expressing cells after treatment with various inhibitors for 18 h. (**B**) Quantification of ECM deposition patterns after treatment with the various inhibitors for 18 h. (**C** and **D**) Levels of mRFPovTSP1C present in conditioned media (CM): (**C**) or total cell lysate (TCL) (**D**) after treatment of transfected cells with the various inhibitors for 18 h. Proteins were resolved on 10% SDS/PAGE gels under reducing conditions. Molecular mass markers are in kDa. In (**A** and **B**), columns represent the mean and bars indicate S.E.M. from three independent experiments. ***P<0.001.

Latrunculin B reduces F-actin levels indirectly, by binding and sequestration of G-actin monomers [40], whereas jasplakinolide stabilizes F-actin and promotes the rate of actin filament nucleation [41,42]. Latrunculin B treatment, although effective for loss of F-actin (Supplementary Figure S1), did not alter the patterning of mRFPovTSP1C puncta (Figures 3A and 3C). However, jasplakinolide treatment, which resulted in cell rounding (Supplementary Figure S1), elevated the proportion of ECM arrays of mRFPovTSP1C that had edge-concentrated puncta, although to a lesser extent than cytochalasin D (Figures 3A and 3C). Treatment of cells with either latrunculin B or jasplakinolide did not alter the proportion of mRFPovTSP1C-expressing cells (Figure 3B).

**Figure 3:**
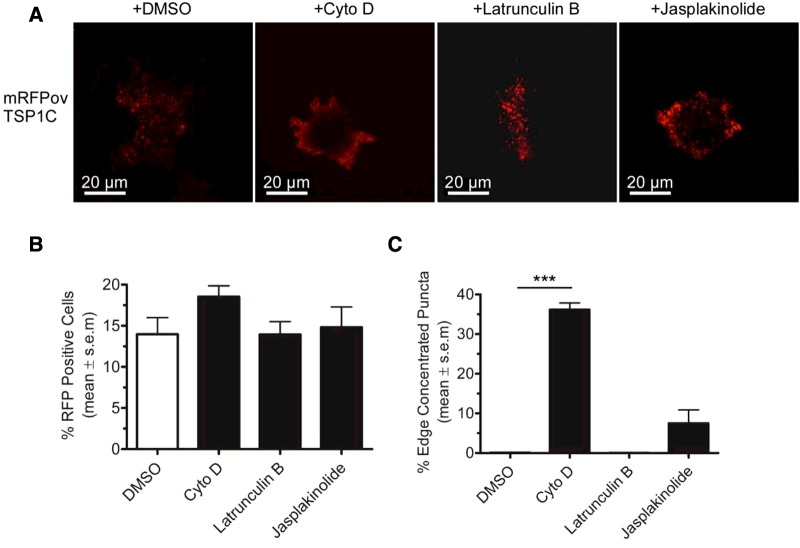
Effect of actin-perturbing inhibitors on ECM patterning of mRFPovTSP1C (**A**) Representative fluorescence images of ECM deposition patterns of mRFPovTSP1C after treatment of cells with vehicle (DMSO), 0.5 μM cytochalasin D, 0.67 μM jasplakinolide or 0.5 μM latrunculin B for 18 h. (**B**) Quantification of mRFPovTSP1C-expressing cells under the different experimental conditions. (**C**) Quantification of edge-concentrated ECM puncta under the different experimental conditions. (**B** and **C**) Columns represent the mean and bars indicate S.E.M. from three independent experiments. ***P<0.001.

Because both cytochalasin D and jasplakinolide act directly on F-actin, albeit by differing mechanisms, these findings indicated that TSP1 puncta patterning might depend on F-actin dynamics. The drug cocktail consisting of jasplakinolide, latrunculin B and Y27632 is reported to be very effective in ‘freezing’ the actin cytoskeleton [43]. This idea was investigated further by testing double or triple combinations of jasplakinolide, latrunculin B and Y27632 for effects on mRFPovTSP1C patterning within the ECM. However, even the triple combination of jasplakinolide, latrunculin B and Y27632 together did not alter ECM patterning of mRFPovTSP1C to a greater extent than jasplakinolide alone (result not shown).

### Relationship of F-actin dynamics to ECM patterning of endogenous thrombospondins

Having established a role of F-actin dynamics in ECM patterning of ectopically expressed mRFPovTSP1C, we next wanted to ask whether the ECM patterning of endogenous TSP also demonstrates a relationship with F-actin dynamics. Pilot experiments determined that SW480 human colon carcinoma cells secrete TSP1 and RCS cells secrete TSP5. Each cell line was tested with a range of cytochalasin D concentrations, to determine the specific conditions under which F-actin organization would be disrupted without preventing cell attachment or reducing cell viability over the time period of the experiment. SW480 cells under control conditions exhibit both round and extended shapes due to their high cell motility [44]. Treatment of SW480 cells with 0.25 μM cytochalasin D over 18 h resulted in a slightly increased fraction of rounded cells (Figure 4A, bright-field images) and a clear alteration of actin organization from microfilaments to aggregates in the cells (Figure 4A, fluorescence panels). In the case of RCS cells, to avoid loss of cells, it was necessary to allow cells to attach for 24 h prior to treatment with 1 μM cytochalasin D. This treatment resulted in clear rounding and reduced spreading of RCS cells (Figure 4A, bright-field panels), with loss of actin microfilaments (Figure 4A, fluorescence panels). Under control conditions, TSP1 is deposited into the ECM of SW480 cells as arrays of puncta; both round and extended shapes of arrays were detected and this probably relates to the range of morphologies of these cells. Examples are shown in Figure 4(B). After treatment of SW480 cells with cytochalasin D, an increase in arrays with edge-concentrated puncta was observed compared with the DMSO controls (Figure 4B). For SW480 cells, the percentage of arrays with edge-concentrated puncta increased from a mean of 8% to a mean of 29% after cytochalasin D treatment (Figure 4C). Under control conditions, RCS cells deposit TSP5 into ECM in the form of arrays of uniformly-spaced puncta (Figure 4D). This patterning was dramatically changed in the ECM of cytochalasin D-treated cells: overall, much less TSP5 was detected in ECM and the arrays that were present all showed large TSP5 puncta at their edges. Multiple examples are shown in Figure 4(D). The smaller size of these arrays probably relates to the much reduced spreading of RCS cells after cytochalasin D treatment. These findings indicate that reorganization of ECM patterning of TSPs upon cytochalasin D treatment is relevant to endogenously-expressed TSPs.

**Figure 4:**
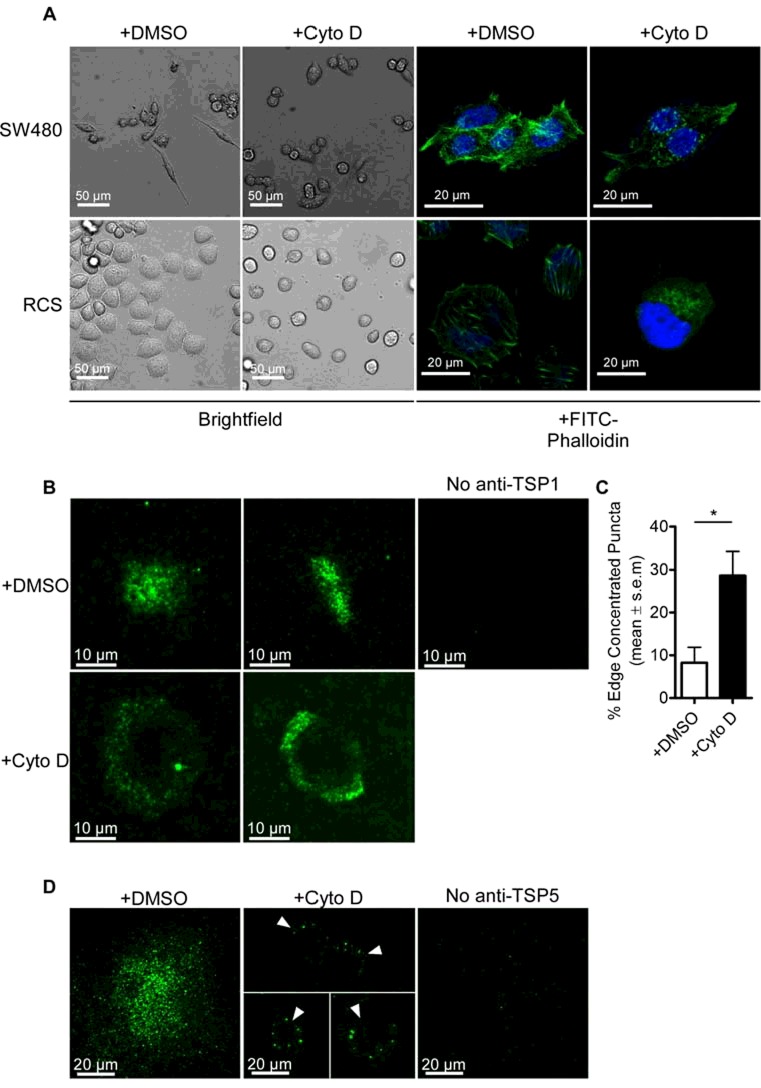
Effect of cytochalasin D treatment on ECM patterning of endogenous TSP proteins expressed by SW480 and RCS cells (**A**) Representative bright-field images and confocal XY sections of SW480 and RCS cells treated with either 0.25 μM (SW480) or 1 μM (RCS) cytochalasin D or DMSO solvent only for 18 h. For confocal microscopy, cells were stained with FITC–Phalloidin (green) and DAPI (blue) prior to imaging. (**B**) Representative fluorescence images of ECM deposition patterns of endogenous TSP1 by SW480 cells, after treatment of cells with 0.25 μM cytochalasin D or vehicle (DMSO) for 18 h. An image of SW480 ECM without primary antibody is included as a control. (**C**) Quantification of edge concentrated ECM puncta of endogenous TSP1 by SW480 cells,±cytochalasin D. (**D**) Representative fluorescence images of ECM deposition patterns of endogenous TSP5 by RCS cells, after treatment of cells with 1 μM cytochalasin D or vehicle (DMSO) for 24 h. For cytochalasin D treated cells, three separate images are shown at the same scale and arrowheads indicate circular ECM arrays. An image of RCS ECM without primary antibody is included as a control. In (**C**), columns represent the mean and bars indicate S.E.M. from four independent experiments. *P<0.05.

### Relationship of F-actin dynamics to ECM patterning of other thrombospondin family members

Having established that F-actin dynamics have physiologically relevant roles in the ECM patterning of TSPs, we examined more widely other TSP family members, including TSPs that assemble in different oligomeric states and non-mammalian TSPs. *Ciona intestinalis* TSPA assembles as a dimer [45] and *Drosophila melanogaster* TSP (D-TSP), like human TSP5, is a pentameric TSP [36,46]. To enable accurate comparison in the experiments, the proteins were expressed under the same conditions in COS7 cells and were detected with the same antibody to the V5 tag. In each case, whereas the majority of ECM puncta were uniformly spaced under control conditions, an increase in ECM arrays with ring-like patterning of puncta were observed after cytochalasin D treatment (Figure 5A). In the absence of V5 antibody no staining was observed (Figure 5A). However, the proportion of ECM arrays with altered puncta patterning differed markedly between dimeric, trimeric or pentameric TSPs. Quantification over multiple independent experiments established that each protein was expressed equivalently and the percentage of expressing cells was not altered after cytochalasin D treatment (Figure 5B). However, the effectiveness of cytochalasin D in bringing about the altered, edge-concentrated patterning of puncta correlated inversely with the oligomeric state of the TSP. Thus, the percentage of arrays of edge-concentrated puncta was highest with dimeric *C. intestinalis* TSPA (mean of 41%) and lowest with the pentameric TSPs (mean of 9% for TSP5 and 13% for D-TSP). Nevertheless, the frequency of edge-concentrated puncta was increased significantly for all the proteins after cytochalasin D treatment compared with the control condition (Figure 5C).

**Figure 5:**
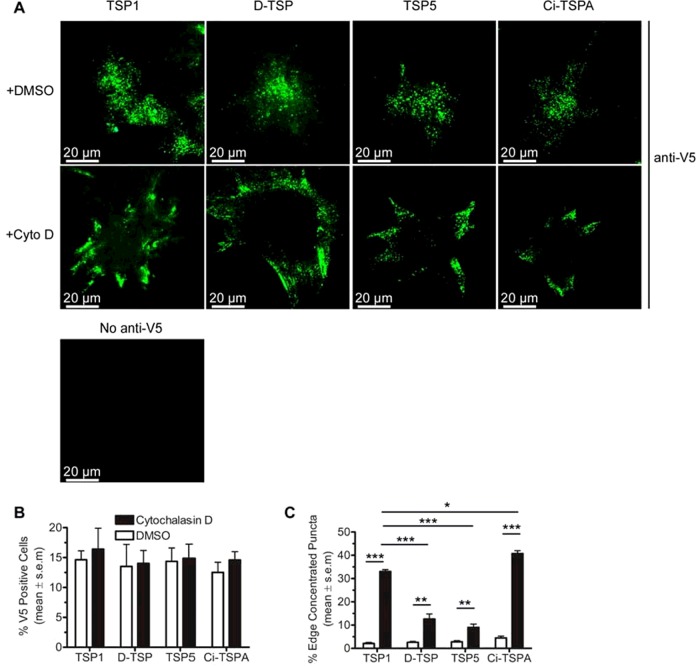
Effect of cytochalasin D treatment on ECM patterning of different TSP family members (**A**) Representative fluorescence images of ECM deposition patterns of V5-tagged TSP1, *Drosophila* TSP (D-TSP), TSP5 or *Ciona* TSPA (Ci-TSPA) after treatment of cells with vehicle (DMSO) or 0.5 μM cytochalasin D for 18 h. An image of TSP1-V5 without primary antibody is included as a negative control. (**B**) Quantification of V5-expressing cells for each protein under the different experimental conditions. (**C**) Quantification of edge-concentrated ECM puncta for each protein under the different experimental conditions. (**B** and **C**) Columns represent the mean and bars indicate S.E.M. from three independent experiments. *P<0.05, **P<0.01, ***P<0.001.

### Effects of altered F-actin dynamics on ECM patterning of an engineered TSP1C oligomer identifies a role of oligomeric state

The above experiments demonstrated a complex relationship between oligomer state and actin modulation and it remained possible that protein sequence determinants, rather than oligomer state, modulate the relationship between F-actin organization and puncta patterning. For example, the L-lectin domain of human TSP1 has 62% sequence identity to that of human TSP5 and 58% identity to the L-lectin domain of D-TSP. To differentiate between the effects of protein sequence and oligomer state, a chimeric TSP was engineered which contained the coiled-coil oligomerization domain from TSP5 fused to the C-terminal region from TSP1. This construct is referred to subsequently as TSP-5-1C (Figure 6A). To control for possible tag-specific effects, constructs with different tags were generated, one with FLAG alone and the other with mRFP and FLAG (Figure 6A).

**Figure 6:**
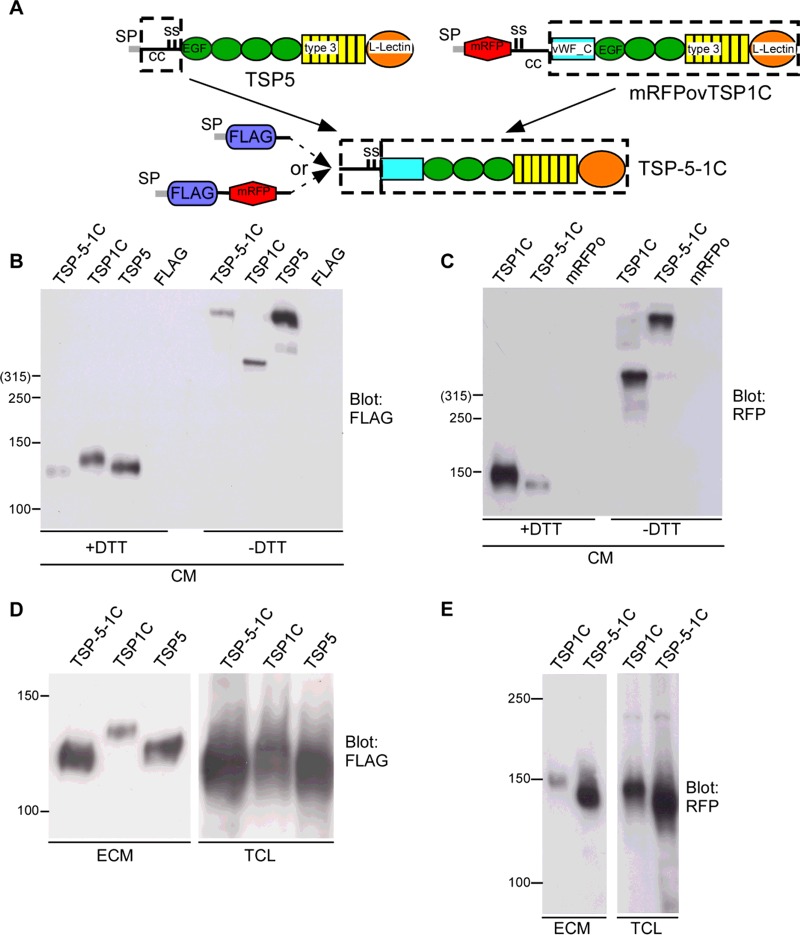
Design and validation of TSP-5-1C chimeric pentamer (**A**) Domain diagrams of TSP5, mRFPovTSP1C and the regions (outlined) used to construct the chimera, TSP-5-1C. FLAG- and FLAG–RFP-tagged constructs were generated. Abbreviations: cc, coiled-coil; EGF, EGF-like domain; L-lectin, L-type lectin domain; SP, signal peptide; SS, position of cysteines that form inter-subunit disulfide bonds; type 3, TSP type 3 repeats; vWF_C, von Willebrand factor_type C domain. (**B** and **D**) Analysis of FLAG-tagged constructs. (**B**) Immunoblots of anti-FLAG immunoprecipitates from conditioned media (CM), resolved on SDS/PAGE gels under reducing or non-reducing conditions. (**D**) Immunoblots of total cell lysates (TCL) and NH_4_OH-isolated ECM from COS7 cells transfected with the indicated constructs. (**C** and **E**) Analysis of FLAG–RFP tagged constructs. (C) Immunoblots of RFP-Trap pulldowns from CM. (**E**) Immunoblots of total cell lysates and NH_4_OH extracted ECM from transfected COS7 cells. (**D** and **E**) Panels are from the same original immunoblot. To improve the resolution of high-molecular mass proteins, proteins below 75 kDa were run off the SDS/PAGE gel; hence, the mRFP protein was not detected in C. (**B**–**E**) All molecular mass markers are given in kDa. The 315 kDa mass marker was from a different protein ladder run on the same gel in a separate lane.

To assess whether these novel TSP-5-1C proteins oligomerized correctly and could accumulate within ECM, these proteins or a suite of control proteins, were expressed transiently in COS7 cells and then isolated from conditioned media, with either RFP-Trap_A beads for the mRFP-tagged proteins or anti-FLAG immunoprecipitation for the FLAG-tagged proteins. Total cell lysates and ECM were also collected. From the conditioned media, each protein migrated at its expected molecular mass when resolved by SDS/PAGE under reducing conditions (Figures 6B and 6C). When resolved under non-reducing SDS/PAGE conditions, the FLAG–TSP-5-1C and mRFP–TSP-5-1C proteins each migrated with higher apparent molecular mass than the respective control TSP1C protein. The apparent molecular mass of FLAG–TSP-5-1C was equivalent to that of the natural pentamer, FLAG–TSP5 (Figure 6B). Equivalent results were obtained if the FLAG–mRFP-tagged proteins were immunoblotted for FLAG instead of RFP (result not shown). Thus, the chimeric proteins are secreted by cells and oligomerize efficiently with the characteristics of a pentamer. Although present within the cell layer at similar levels to the control proteins, FLAG–TSP5-1C was typically present in conditioned media at lower levels than the FLAG–TSP1C or FLAG–TSP5 control proteins. A similar trend was noted for mRFP–TSP-5-1C (Figures 6B and 6C).

Having established that the TSP-5-1C proteins oligomerize and are secreted effectively, ECM was prepared from transfected COS7 cells and extracted in hot SDS/PAGE sample buffer under reducing conditions [20]. Both FLAG–TSP-5-1C and mRFP–TSP-5-1C were detected in the ECM (Figures 6D and 6E). Interestingly, both proteins accumulated within ECM to a much higher level that the respective trimeric TSP1C (Figures 6D and 6E). We infer that the lower levels in media may relate to the very strong accumulation in ECM.

Next, the patterning of these proteins in ECM and the effects of cytochalasin D were examined by fluorescence microscopy. FLAG–TSP-5-1C displayed a similar ECM patterning to FLAG–TSP1C in the absence of cytochalasin D, with distinct fluorescent puncta distributed quite uniformly in the ECM arrays (Figure 7A). In the absence of FLAG antibody, no staining was observed (Figure 7A). A similar uniform distribution of puncta was observed for mRFP–TSP-5-1C in comparison with mRFPovTSP1C (Figure 7A). For both the TSP-5-1C proteins, the puncta tended to be denser within the arrays than for the trimeric TSP1C (Figure 7A). The percentage of cells expressing each recombinant protein was not altered significantly after cytochalasin D treatment (Figures 7B and 7D). However, cytochalasin D treatment resulted in a significant increase in ECM arrays with puncta concentrated at the edges for all proteins in comparison with the control conditions (Figures 7C and 7E). Furthermore, the occurrence of edge-concentrated puncta was significantly higher for trimeric mRFPovTSP1C or FLAG–TSP1C than for the engineered pentamers (Figures 7C and 7E). These results demonstrate that the oligomeric state of the TSP C-terminal region from TSP1 can determine the efficiency of its accumulation within ECM. The results also show that oligomer state impacts on the responsiveness of ECM patterning to blockade of F-actin dynamics by cytochalasin D.

**Figure 7:**
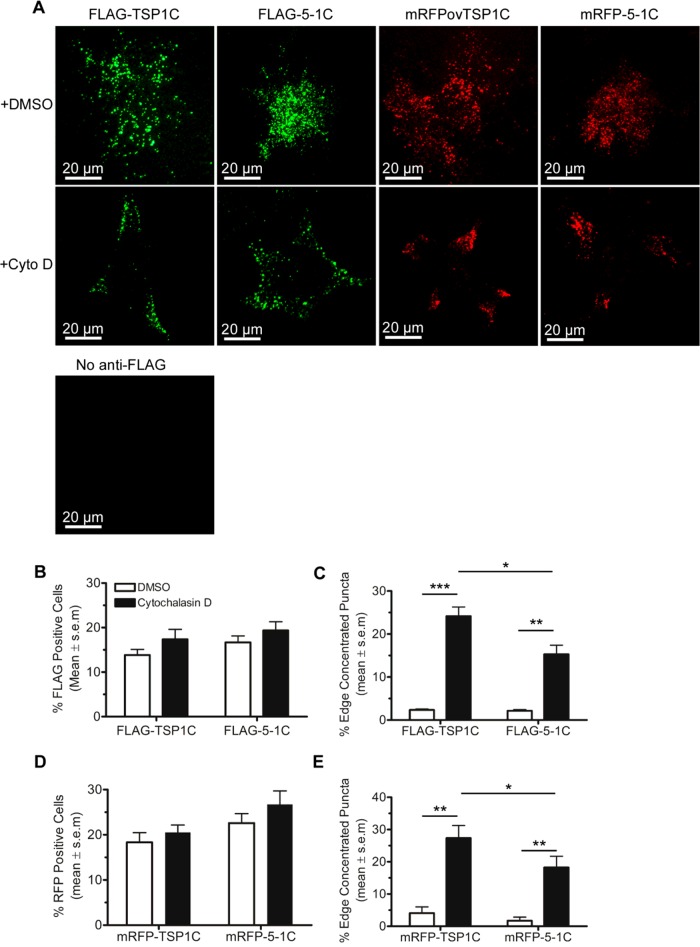
Effect of cytochalasin D treatment on ECM patterning of TSP1C proteins of different oligomer state (**A**) Representative fluorescence images of ECM deposition patterns of TSP1C or TSP-5-1C, after treatment of cells with 0.5 μM cytochalasin D or vehicle (DMSO) for 18 h. Shown for both FLAG- and RFP-tagged proteins. An image of FLAG–TSP1C without primary antibody is included as a control. (**B** and **D**) Quantification of expressing cells for FLAG- (**B**) or RFP-tagged (**D**) constructs±cytochalasin-D. (**C** and **E**) Quantification of edge concentrated ECM puncta for FLAG–TSP1C (**C**) or mRFP–TSP-5-1C (**E**),±cytochalasin D. (**B**–**E**) Columns represent the mean and bars indicate S.E.M. from three independent experiments. *P<0.05, **P<0.01, ***P<0.001.

## DISCUSSION

We present here, data in support of a novel concept whereby F-actin dynamics modulate the patterning of TSPs in the ECM and oligomer state of the TSP is a key determinant in this process. We demonstrate that the ECM patterning of TSPs changes from uniform to edge-concentrated arrays of puncta after treatment of cells with cytochalasin D. This effect was also seen for endogenous TSP1 and TSP5, in support of a physiologically relevant role for F-actin dynamics. Under matched experimental conditions of ectopic expression in COS-7 cells and detection by a V5 epitope tag, this effect was seen for human TSP1 and TSP5, *Drosophila* TSP and *Ciona* TSPA, thus encompassing dimeric, trimeric and pentameric oligomers and evolutionarily distant forms of TSPs. This implicates a conserved mechanism by which global F-actin cytoskeletal dynamics modulate the spatial characteristics of ECM deposition of TSPs. The experiments in COS7 cells also identified that the magnitude of the effect of cytochalasin D treatment on ECM patterning correlates inversely with TSP oligomer state. This was investigated definitively by use of a novel, pentameric chimera between the coiled-coil oligomerization domain of TSP5 and the TSP1 C-terminal region that enabled us to distinguish between effects of protein sequence and oligomer state. The results obtained demonstrate that oligomer state is a key determinant of F-actin-dependent ECM patterning modulation.

Of the pharmacological inhibitors of the F-actin cytoskeleton tested, cytochalasin D had by far the strongest effect, whereas jasplakinolide had a mild effect and latrunculin B had none. Cytochalasin D binds the barbed ends of actin filaments inhibiting both polymerization and depolymerization [37], through a similar mode of action to jasplakinolide which binds to the side of actin filaments [41,42]. In addition, cytochalasins have been shown to bind actin monomers and dimers, altering the kinetics of actin filament growth and slowing elongation [47,48]. This effect is mimicked by latrunculin B [40]; however, latrunculin B did not alter the patterning of mRFPovTSP1C within ECM. Taken together, these results suggest that the combined actions of cytochalasin D on binding to F-actin barbed ends and modulating F-actin dynamics by altering the nucleation pool available for microfilament elongation is the most likely reason for its striking effect on the ECM patterning of TSPs.

With regard to ECM retention of TSPs as a cell-mediated process, we note interesting differences in the role of the actin cytoskeleton compared with structural ECM components such as fibronectin. In the case of fibronectin matrix assembly, Rho-dependent, actomyosin-based contractility is essential for conformational changes that enable fibrillogenesis from cell-surface bound fibronectin dimers [30–32]. In contrast, the patterning of TSP puncta depended strongly on polymerized actin but was not markedly altered by changes to actomyosin contractility or Rho-dependent signalling. A specific role of the Rho pathway in puncta patterning was not detected; however, perturbation of the Rho pathway did decrease the secretion of mRFPovTSP1C.

The differential responsiveness of natural TSPs of different oligomer states to altered F-actin dynamics may relate to the different tissue contexts and expression patterns of TSP family members [1]. TSP5 is expressed throughout life in cartilage and tendon, tissues that undergo major deformation and in which cells are exposed constantly to a range of tensile stresses [1,49,50]. Similarly, D-TSP is present at muscle–tendon attachment sites [51,52]. In contrast, TSP1 is at very low levels in most adult tissues and becomes elevated in ECM in situations of tissue remodelling or fibrosis, in which F-actin cytoskeletal organization in cells may be altered or undergoing dynamic changes as cells migrate [21]. For example, TSP1 is elevated in atherosclerotic plaques and is an inducer of smooth muscle cell migration and proliferation [33,53–55]. The greater sensitivity of ECM patterning of trimeric TSP1 to altered F-actin dynamics may assist the co-ordination of changes in cell behaviour with spatiotemporal variations in ECM microenvironment.

The inverse correlation between responsiveness of ECM patterning to altered F-actin dynamics and higher order oligomer state of TSPs was investigated definitively by use of a chimeric construct that fused the coiled-coil oligomerization domain from TSP5 with the C-terminal region of TSP 1. This increase in oligomer state sharply increased the level of ECM deposition (Figures 6D, 6E and 7A). In contrast, the effect of cytochalasin D treatment on the ECM patterning of TSP-5-1C was decreased in comparison with mRFPovTSP1C. So, whereas the chimeric protein accumulates in ECM to a greater extent, the influence of F-actin dynamics on ECM patterning is diminished, representing two separate facets of the functional significance of oligomer state over sequence-specific activity for TSPs. All experiments using the chimera were performed with two different tagged constructs, reducing the possibility of tag-specific artifacts. We note that a contribution of protein sequence to the functional response is not excluded, since pentameric TSP5 showed more limited alteration of patterning than D-TSP (Figure 4). The results implicate that a common, actin-dependent, modulatory mechanism can function in distinct ways for the different forms of TSPs.

In future, it will be of interest to examine further the cellular mechanisms that lead to edge-concentration of TSP puncta arrays within ECM, in particular whether this relates to spatially-restricted trafficking and secretion of TSP-containing vesicles, specific aspects of the association of vesicles with F-actin or to the localization of cell-surface or ECM-located TSP-binding proteins. The TSP-5-1C chimeras will be useful tools to investigate the influence of sequence compared with oligomer state for the range of TSP1 functions that span from effects on cell phenotype and migration to ECM organization.

## Abbreviations

ECM: extracellular matrix
LPA: lysophosphatidic acid
mRFP: monomeric RFP
RCS: rat chondrosarcoma
TSP: thrombospondin
